# Audio-visual integration is more precise in older adults with a high level of long-term physical activity

**DOI:** 10.1371/journal.pone.0292373

**Published:** 2023-10-04

**Authors:** Zahra Azizi, Rebecca J. Hirst, Fiona N. Newell, Rose Anne Kenny, Annalisa Setti

**Affiliations:** 1 School of Psychology and Institute of Neuroscience, Trinity College Dublin, Dublin, Ireland; 2 The Irish Longitudinal Study on Ageing, Trinity College Dublin, Dublin, Ireland; 3 School of Applied Psychology, University College Cork, Cork, Ireland; 4 Mercer Institute for Successful Ageing, St. James Hospital, Dublin, Ireland; University of New England, AUSTRALIA

## Abstract

It is well established that physical activity leads to numerous health, cognitive, and psychological benefits. However, to date, very few studies have investigated the impact of physical activity on multisensory perception, that is, the brain’s capacity to integrate information across sensory modalities. Furthermore, it is unknown what level of long-term physical activity is associated with multisensory integration in adults. We explored the relationship between multisensory integration and a ten-year physical activity trajectory in 2,974 adults aged 50+ from The Irish Longitudinal Study on Ageing by measuring susceptibility to the Sound Induced Flash Illusion (SIFI) at multiple audio-visual temporal asynchronies. Physical activity was measured using the International Physical Activity Questionnaire (IPAQ-SF) at 2 years intervals over ten years. We used latent class trajectory modelling to identify latent growth classes of individuals following a similar trajectory of physical activity over time. We analysed the association of this trajectory with performance accuracy to the illusion trials of the SIFI task with generalized logistic mixed effects regression models, adjusted for several covariates. Results showed that more precise integration (i.e., lower SIFI susceptibility with larger temporal asynchronies) was associated with a higher level of sustained physical activity across ten years. Although the use of self-reported physical activity and a short version of the SIFI task limit our conclusions to some extent, nonetheless, the results suggest that sustained physical activity is associated with more precise multisensory integration, which in turn is linked to better balance and a lower risk of falling in older adults.

## Introduction

The perception of our world requires us to integrate inputs from various sensory modalities (e.g., visual, auditory, tactile, etc.). Our ability to carry out this process does not remain static across the lifespan, and the pattern of multisensory integration changes with ageing (for review see [1]). It has been demonstrated that older adults perceive and respond to multisensory stimuli differently than younger adults [2–4]. For instance, older adults experience some multisensory perceptual illusions more frequently than younger control participants [3, 5–8].

At present, it is unclear whether all changes in processing multisensory stimuli are beneficial for older adults. For example, more pronounced multisensory enhancement in older adults (i.e., reaction time enhancements when audio-visual stimuli are presented simultaneously) is associated with a lower risk of falls and better cognition [9]. However, older adults also appear to integrate information across longer temporal intervals, suggesting a larger Temporal Binding Window (TBW). The TBW refers to the temporal duration during which the integration of auditory and visual signals is most likely to occur (see e.g., [3]). The role of the TBW in multisensory integration can be demonstrated using an illusion known as the Sound Induced Flash Illusion (SIFI). The illusion is experienced when the observer reports seeing two flashes accompanied by 2 beeps when only one flash is actually presented. Susceptibility to SIFI is dependent on the stimulus onset asynchrony (SOA) between the accompanying flash-beep pair and second beep stimuli. Thus, using the accuracy of performance to the SIFI [10, 11], it was shown that a narrower temporal TBW is more typical of younger adults, while older adults are more susceptible to the SIFI illusion at longer SOAs suggesting a larger TWB in this age group [8]. This wider window has been associated with poorer cognitive trajectories [12, 13], fall risk [8, 14], and poorer balance in ageing [15].

There is compelling evidence to support the idea that physical activity (PA) level and multisensory integration processes in older adults are inextricably linked. Changes in the visual, somatosensory, proprioceptive, and vestibular systems are common as we age. These changes limit our sensory capabilities, and as a result, there may be fewer sensory and motor resources available to sustain adequate postural control [16–19], and gait [20]. Furthermore, these resources are also likely required for more complex motor activities such as physical activity [21]. Results from Prioli [19], who investigated the relationship between sensory integration, postural control, and physical activity in older adults, suggest that sedentary older adults have more difficulty integrating sensory information than active older adults and younger adults and that physical activity may improve sensory integration. Correspondingly, there are beneficial effects of engaging in physical activities [6] and more specifically open-skill exercise (i.e., exercises in a dynamic and unpredictable environment [e.g., table tennis]) [22] on multisensory perception. Therefore, on the one hand, efficient multisensory integration could be a pre-condition, or a facilitator of physical activity, whereas, on the other hand, performing physical activity could improve multisensory processing. O’Brien [22] showed that one bout of open-skill exercise could make multisensory perception more precise. The authors argued that open-skill exercise could provide multisensory training, similar to what has been shown for cognitive performance, particularly executive functions. However, in that study there was no significant correlation between the amount of regular physical activity, measured by the International Physical Activity Questionnaire (IPAQ), and multisensory perception, leaving open the question of whether the effect was mainly due to a temporary change in arousal.

Physical activity is known to play an important role in the protection against cognitive decline and dementia [23–25]. Physical activity intervention studies in older adults have demonstrated effects on brain structure, function, and neural connectivity [26–30]. While preliminary evidence indicates that leisure time physical activities with higher intensity seem to be of particular relevance for maintaining cognitive function in old age [31–33], information concerning the specific impact of light-intensity physical activity is currently missing. Furthermore, while cross-sectional analysis [2, 13] and a longitudinal analysis involving older adults [12] have demonstrated that multisensory integration is sensitive to cognitive performance, to date no studies of multisensory perception in ageing have explored the association between multisensory integration and longitudinal physical activity trajectories. Longitudinal trends in physical activity research may be beneficial in determining whether multisensory integration is sensitive to long-term physical activity in ageing populations.

The current study aimed to address the current gap in knowledge of how multisensory integration and physical activity are associated over the long term by involving a large sample of older adults drawn from The Irish Longitudinal Study on Ageing (TILDA) [34]. The accuracy of performance on the SIFI task [10, 11] was used as a valid and reliable measure of audio-visual integration. In order to comprehensively capture the physical activity trajectory, we assessed self-reported physical activity, based on IPAQ reported at each testing wave [35] of the TILDA protocol: longitudinal trajectories were therefore constructed for IPAQ level incorporating data spanning ten years (i.e., five testing waves). The IPAQ is a widely utilised measure of physical activity [36]. If multisensory integration is linked to long-term patterns of physical activity, we would expect older adults who have been consistently active for a long time (i.e., over ten years) to have more precise temporal multisensory integration, i.e., a narrower TBW, indicated by lower susceptibility to the SIFI at longer Stimulus Onset Asynchronies (SOAs), than their less active counterparts.

## Methods

The Irish Longitudinal Study on Ageing (TILDA) is a large prospective cohort study that looks at the social, economic, and health conditions of people living in the Republic of Ireland who are 50 years of age or older. TILDA began collecting data in 2009 with successive data collection cycles every two years. Five waves of data from TILDA were available at the time of the current analysis. The sample was drawn from a clustered random sample of all households in the Republic of Ireland. Surveyors called addresses on a list to see if the occupants complied with the criteria for the study, which included being at least 50 years old and dementia-free. A computer-assisted personal interview (CAPI) administered at home, a self-completion questionnaire (SCQ), and a physical and cognitive health assessment run by professional research nurses in the TILDA centres were all used to gather the data for waves 1 and 3, whereas the data for waves 2, 4, and 5 only included the CAPI and SCQ (i.e., no in-centre health assessment). Wave 3 included our primary measure, performance to the SIFI test and, the full IPAQ assessment from all five waves was utilized. A more detailed description of the TILDA study’s general design and protocol has been reported elsewhere [34].

### Participants

**Fig 1:**
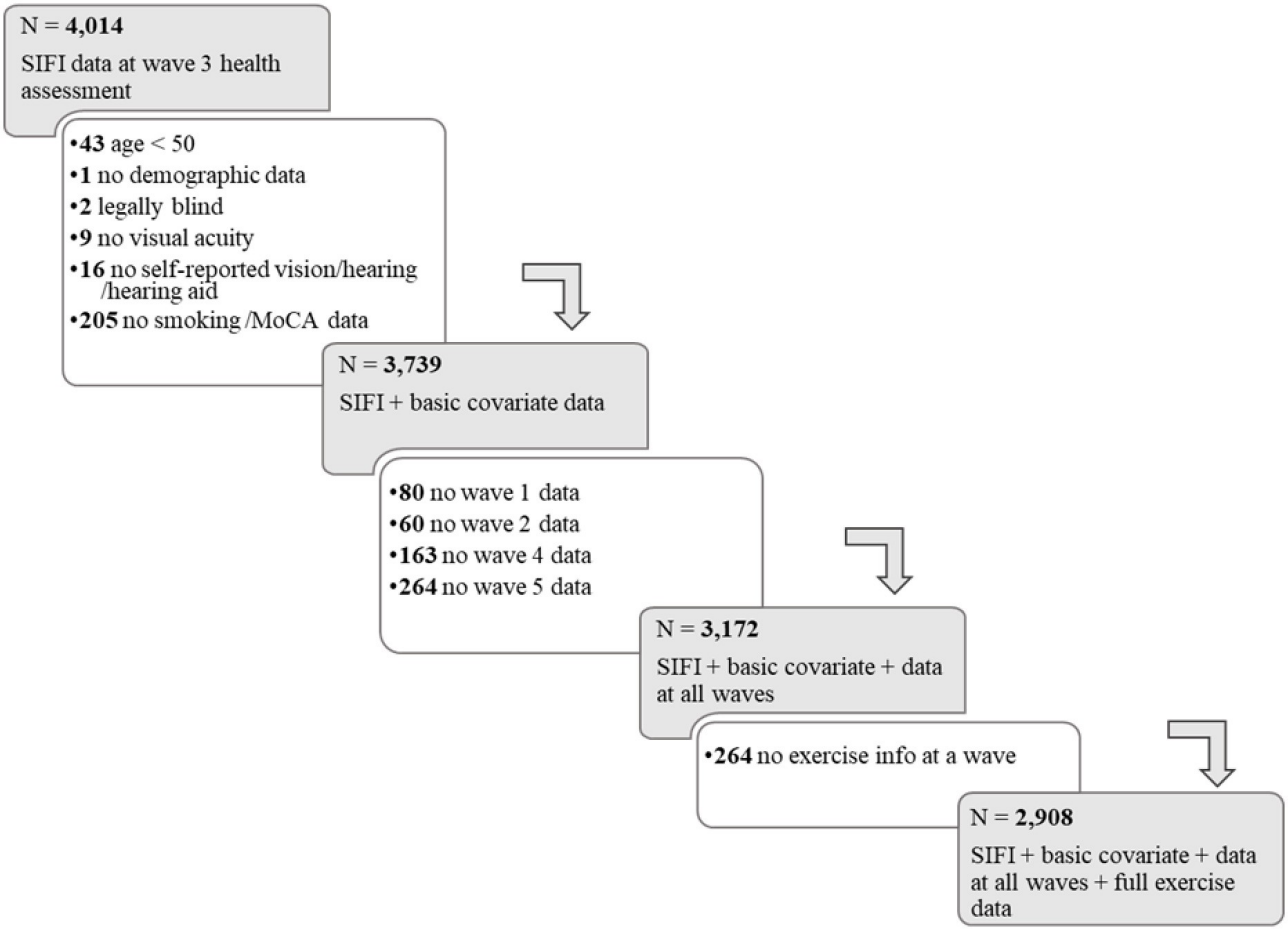
Protocol for selecting participants for this study from TILDA.

Participants were drawn from Waves 1–5 (2009–2018) of TILDA. Fig 1 depicts the general inclusion/exclusion criteria, and Table 1 displays the demographics of the sample that was included in the study. Consistent with previous studies involving the TILDA cohort, in which SIFI susceptibility was the outcome measure [2, 12], participants must have completed the SIFI at wave 3 of TILDA alongside a full health assessment (4,014). Participants who did not partake in any other waves (n = 672), were younger than 50 years (n = 43), registered as legally blind (n = 2), and/or missing data for other key measures (n = 323) were omitted prior to analysis. The Trinity College Faculty of Health Sciences Ethics Committee approved the study, the testing procedures followed the Helsinki Declaration, and data collection, storage, and processing followed GDPR guidelines. All participants provided written, informed consent at all the waves.

**Table 1:** Descriptive characteristics of the sample.

		All Sample (n = 2,974)	Males (n = 1,316)	Females (n = 1,658)
Age	Min	50	50	50
	Max	89	87	89
	Mean (sd)	64.51 (7.77)	65.15 (7.78)	63.99 (7.71)
Physical Activity Trajectory	Stable [Low] (%)	782 (26)	256 (19)	526 (32)
	Decreasing (%)	967 (33)	406 (31)	561 (34)
	Stable [High] (%)	766 (26)	438 (33)	328 (20)
	Increasing	459 (15)	216 (16)	243 (15)
Education	Primary/none (%)	477 (16)	251 (19)	226 (14)
	Secondary (%)	1,182 (40)	528 (40)	654 (39)
	Third/higher (%)	1,315 (44)	537 (41)	778 (47)
BMI	Min	16.83	17.04	16.83
	Max	44.20	44.20	43.97
	Mean (sd)	28.54 (4.57)	28.85 (4.11)	27.84 (4.87)
Smoking history	Never smoked (%)	1,441 (48)	541 (41)	900 (54)
	Past smoker (%)	1,258 (42)	655 (50)	603 (36)
	Current smoker (%)	275 (9)	120 (9)	155 (9)
Heavy drinker	No (%)	2,303 (86)	1,092 (83)	1,459 (88)
	Yes (%)	373 (14)	224 (17)	199 (12)
MoCA score	Min	9	9	12
	Max	30	30	30
	Mean (sd)	26.28 (2.87)	26.11 (2.97)	26.31 (2.85)

### Sound-Induced Flash Illusion

**Fig 2:**
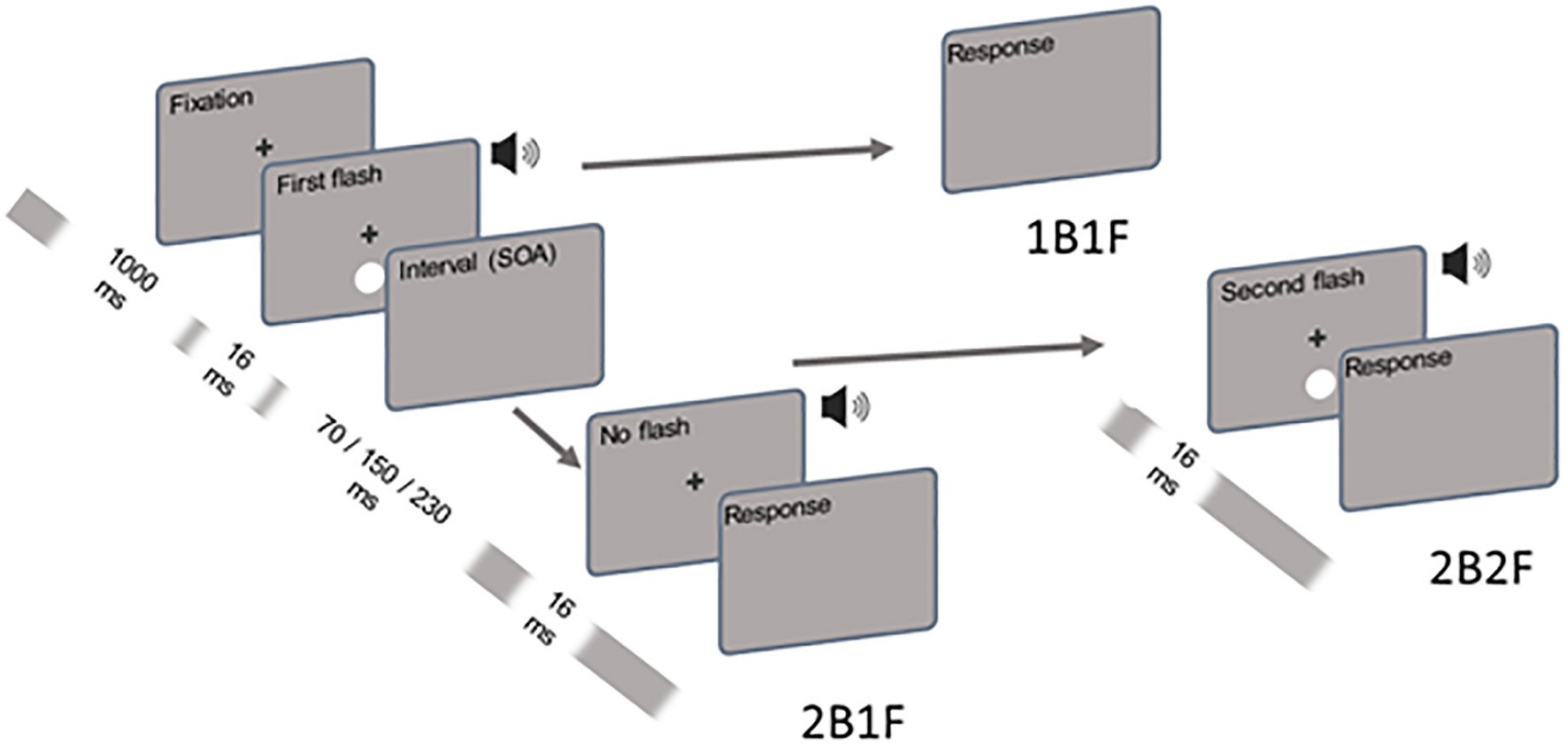
A schematic illustration of the Sound Induced Flash Illusion task. During a trial of the SIFI task, the visual stimulus (white circle) was displayed below the fixation cross, while the pure tone auditory beep was provided. The trials were either congruent or illusory. Congruent trials consisted of a single flash-beep pair (1B1F), or two consecutive flash-beep pairs (2B2F) separated by a variable stimulus onset asynchrony (SOA). A flash-beep pair was first shown during the illusory trials, followed by a second auditory beep (2B1F) at some SOAs (left panel). Participants were asked how many flashes they perceived during the trial, regardless of trial type.

The SIFI is a common example of an auditory-dominated multisensory integration phenomenon in which the simultaneous presentation of a different number of auditory beeps causes the observer to misjudge the number of visual stimuli (or "flash") [3]. The SIFI test was included as part of a comprehensive health assessment at wave 3 of TILDA only. The stimuli used were those used in earlier studies [37]. See Fig 2 for an illustration of the different trial times used in the SIFI task. The visual and/or auditory stimuli were presented after a fixation cross lasting 1000 ms marked the start of each trial. The visual stimulus was a white disk that was projected onto a black background 5 cm below the central fixation cross for 16 milliseconds. Its luminance was about 32 foot-lambert and its visual angle was about 1.5°. Each auditory "beep" stimulus consisted of a short burst of 3,500-Hz noise (10 ms, 1 ms ramp), with a volume of about 80 dB. The participants were instructed to verbally report the number of flashes and/or beeps perceived in each trial. The main testing block contained multisensory non-illusory trials (2B2F, 1B1F), multisensory illusory trials (2B1F), and unisensory visual trials (0B2F, 0B1F). Illusory trials (2B1F) were presented at one of six Stimulus Onset Asynchronies, −230 ms, −150 ms, −70 ms, 70 ms, 150 ms, and 230 ms, where negative values indicate an auditory lead. While unisensory 0B2F trials were presented at one SOA of 70 ms, congruent 2B2F trials were existing at three SOAs, 70 ms, 150 ms, and 230 ms. Due to time constraints, a total of twelve trials were completed per experimental condition (i.e., two trials per SOA across pre-post conditions). Additional details on the SIFI protocol within TILDA are described elsewhere [38].

### IPAQ

Physical activity was assessed at all waves using the eight-item short form of the International Physical Activity Questionnaire (IPAQ) [35, 39] Participants reported the number of days and duration of vigorous, moderate, and walking activities that were undertaken during the previous seven days. Data were summed within each activity category (i.e., vigorous intensity, moderate intensity, and walking) to estimate the total number of minutes engaged in physical activity per week. Respondents who reported walking or moderate-to-vigorous physical activity greater than a combined 16 h/day and those who reported more than 7 days active during a week were excluded as these responses were considered unreasonable [40]. The remaining participants were classified according to whether they met World Health Organization (WHO) physical activity guidelines, based on weekly metabolic equivalents (MET). METs are multiples of the resting metabolic rate that are yielded as a score in MET. min. A MET. min is computed by multiplying the MET score by the minutes performed. The selected MET values for each type of activity were derived from work undertaken during the IPAQ Reliability Study (Walking = 3.3 METs, Moderate PA = 4.0 METs, and Vigorous PA = 8.0 METs; Craig et al., 2003). Based on weekly MET. min of PA, participants were divided into tertiles, categorized into low (0 to <600 MET. min), moderate (600 to <1200 MET. min), or high (≥1200 MET. min) doses according to standard guidelines (www.ipaq.ki.se).

### Statistical analysis

We used the R statistical programming environment, version 4.2.0 [41] for all analyses. Measures of physical activity were available for five waves of the TILDA study (providing 10 years’ worth of data per participant).

#### The trajectory of physical activity

To identify trajectories of physical activity over time, we used latent class trajectory modelling (LCTM), which is a specialized form of finite mixture modelling designed to identify latent growth classes of individuals following a similar progression of a determinant over time [42]. This statistical method for longitudinal data classifies study participants (i.e., groups) based on their initial level and patterns of change over time [43]. In the current study, this was carried out with the ’lcmm’ packages in R [42]. Criteria to determine the best-fitting number of trajectories included (i) minimum Bayesian Information Criterion (BIC) (see Supplementary Materials) (ii) overall meaning within the data (iii) class size (at least 5% of the population) [42, 44].

#### Association of physical activity trajectory with multisensory integration

To explore the relationship between physical activity trajectories and multisensory integration at wave 3, the identified classes were included as predictors in a generalised logistic mixed effects model with accuracy for judging the number of flashes to illusion trials on the SIFI (2 beeps and 1 flash) as an outcome measure. We used generalized logistic mixed-effect models using “glmer” in the “lme4” package (family = “binomial”) [45]. Stimulus Onset Asynchrony (SOA; 70, 150, or 230 ms), physical activity trajectories, and any interactions between SIFI SOA and physical activity (indicatively showing various patterns of multisensory integration based on physical activity) were fixed effects of interest. A random effect was retained for participant ID. All continuous, numeric variables were scaled and centered using the "scale ()" function in R before being incorporated into the model. The model is reported adjusted for the following factors: (i) Demographic: age, sex of the participant (male/female), the education level (Primary, Secondary, Third/Higher); (ii) Health: chronic conditions (0, 1 or 2 + of the following: Parkinson’s disease, lung disease, asthma, arthritis, cancer, osteoporosis), cardiovascular conditions (0, 1, or 2 + of the following: TIA, stroke, heart attack, heart murmur, heart rhythm, angina), depression (Yes or No based on a CESD score of > = 9), Body Mass Index (BMI; kg/m2), and cognitive assessment score based on Montreal Cognitive Assessment (MoCA); (iii) Lifestyle: respondent’s social connectedness score (count of the following: a member of organisation or church, married/ living with partner as if married, has at least one close relative or friend), smoking (never smoked, past smoker, current smoker), and problematic alcohol consumption (no/ yes) based on CAGE questionnaire where a score ≥ 2 indicated problematic drinking; (iv) Sensory: self-reported vision and self-reported hearing (Excellent, Very Good, Good, Fair, Poor), Visual Acuity Score (VAS = 100–50 × LogMAR, so that a VAS of 100 represents a LogMAR score of 0 (20/20 vision), higher scores therefore indicate better acuity). We adjusted additionally for accuracy for judging 2 unisensory beeps (2B0F) at 70 ms, accuracy for judging 2 flashes alone (0B2F) at 70 ms, and accuracy for judging the number of flashes when 1 beep and 1 flash were presented together (1B1F) in the model to take account of differences in unimodal visual and unimodal auditory trials. Our models also controlled for stimulus order, i.e., whether the second beep preceded/led or followed the flash beep pair (termed “Pre/Post” respectively) and included MoCA*SOA and Pre-Post*SOA interaction terms.

To answer our primary research question, which was whether physical activity trajectory was associated with susceptibility to the SIFI at longer SOAs, we tested whether the physical activity trajectory classes significantly interacted with SIFI SOA. The significance of the physical activity trajectory class by SOA interaction term was assessed using the likelihood ratio test to compare the model with the interaction term to the model without the interaction term (the additive model) using the “anova ()” function in R. Moreover, we examined cross-sectional relationships between multisensory perception and physical activity using the IPAQ level of participants at the same time point as the SIFI (i.e., at wave 3).

## Results

**Fig 3:**
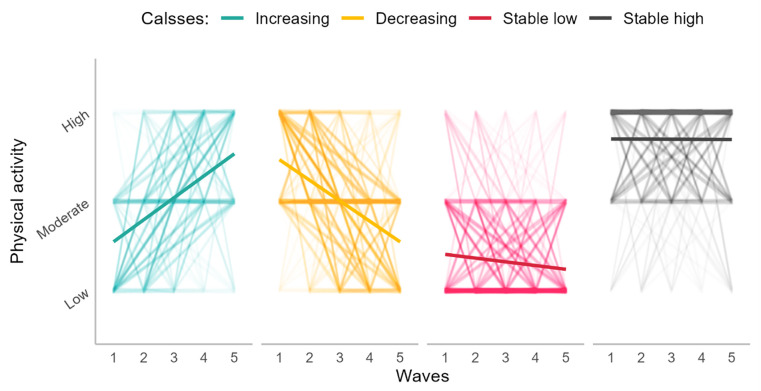
Cluster trajectory classes for PA levels. Classes were labelled as “Increasing”, “Decreasing”, “Stable [Low]”, and “Stable [High]” reflecting the physical activity level trajectories across five waves (i.e., ten years).

Table 1 describes the characteristics of the sample. The mean age of the sample was 64.51 years (SD = 7.7), and 44.2% were male. A solution of four classes was selected as optimal for IPAQ trajectory considering five waves based on BIC (see S1 Fig and S1 Table) and the size of classes. The four classes consisted of those with a relatively high IPAQ level across all waves (stable high) (n = 766 (%26); high physical activity), a relatively low level of IPAQ across all waves (stable low) (n = 782 (%26); low physical activity), increasing IPAQ levels (n = 459 (%15); increasing physical activity), or declining IPAQ levels (n = 967 (%33); decreasing physical activity) over ten years as shown in Fig 3 below. More details on these four classes are in S2 Table.

**Fig 4:**
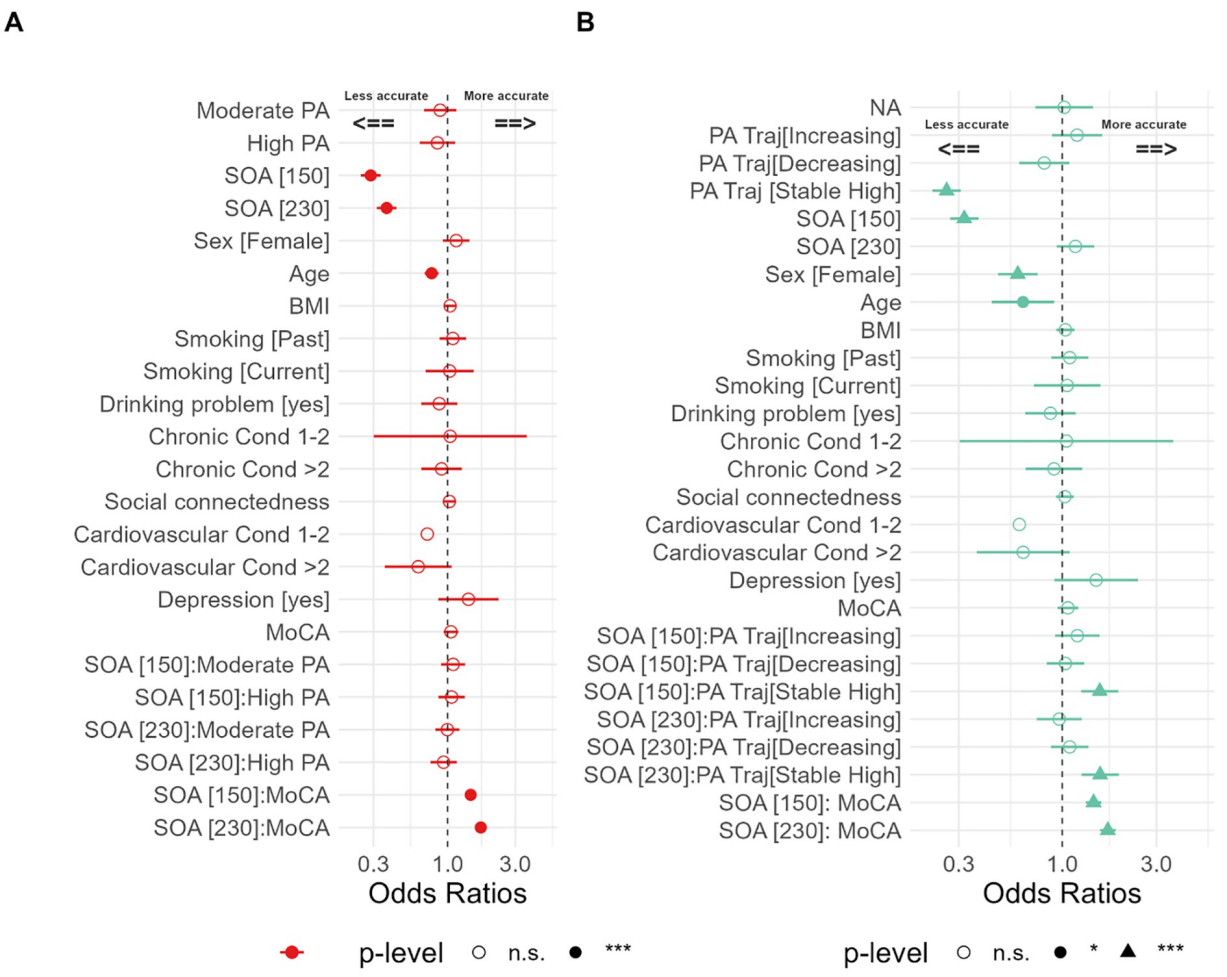
Odds ratio (OR) for making a correct response on illusory 2B1F trials (i.e., a “one flash” response) with 95% confidence intervals based on predictors. Odds ratios to the left of the dashed line signify the reduced odds of making an accurate response (i.e., increased illusion susceptibility), and odds ratios to the right of the dashed line signify the increased odds of making an accurate response (i.e., reduced illusion susceptibility). **(A)** Cross-sectional model. **(B)** Longitudinal model, the odds ratios for interactions between SOA and PA level trajectory in the “Stable [High]” can be seen.

A summary of the results from the cross-sectional model is shown in Fig 4A and S3 Table. Participants were less accurate (i.e., more susceptible) to the SIFI task on longer SOAs (i.e., 150 and 230 ms). Of note, the PA level had no impact on the SIFI cross-sectionally, and there was no evidence of an interaction with the SOA, nor a main effect. Additionally, older participants were less accurate on the SIFI (i.e., more susceptible) (odds ratio = 0.75, p<0.001, 95% CI [0.68, 0.83]), while MoCA score interacted with SOA, showing that participants with higher cognitive abilities were more accurate on the SIFI at longer SOAs (Fig 4A; S3 Table), which replicates previous results [2].

In general, we will use the terms “Stable [Low]” class, “Decreasing” class, “Increasing” class, and “Stable [High]” class to refer to classes respectively across IPAQ PA levels trajectories. IPAQ trajectory classes were then used as predictors in logistic mixed-effect models with the stable low activity trajectory class (Stable [Low]) set as the reference in the model. The reason for using Stable [Low] as the reference in the model was simply for ease of visualization and interpretation; however, the results remained consistent when other classes were used as the reference. we examined the effect of SOA on the accuracy, using 70 ms as the reference condition. A summary of the results from the longitudinal model is shown in Fig 4B. PA trajectory interacted with longer SOA for ‘Stable [High]’, showing that participants classified in the high physical activity trajectory class across five waves were more accurate on the SIFI at longer SOAs (SOA [150]: odds ratio = 1.37, p = 0.004, 95% CI [1.11, 1.69]; SOA [230]: odds ratio = 1.46, p<0.001, 95% CI [1.18, 1.80]). A likelihood ratio test comparing the full model to a model without the IPAQ trajectory by SOA interaction term indicated that the inclusion of this term significantly improved the model fit (χ^2^(9) = 34.50, p = < 0.001). This model also included the effects of age (older participants had lower SIFI accuracy) and MoCA score interaction with SOA (higher MoCA score had higher accuracy on longer SOAs) (Fig 4B; S4 Table).

## Discussion

This study aimed to examine the association between the precision of multisensory perception and physical activity levels across ten years, measured from repeated assessments utilising the IPAQ in a large sample of adults aged over 50 years. The results showed an association between high levels of sustained physical activity that was stable over 10 years and precise multisensory function, assessed using the SIFI. A high level of physical activity on the IPAQ means that physical activity levels equate to at least one hour of moderate-intensity activity per day or vigorous-intensity activity on at least 3 days per week. As predicted, those in the stable high physical activity, i.e., those who had weekly MET. Min ≥1200, stable-high class were more accurate (i.e., less susceptible) to the SIFI at longer SOAs. This suggests that people who have been regularly active over a long period (i.e., ten years) have more precise temporal multisensory integration (a narrower TBW), as demonstrated by a decreased susceptibility to the SIFI at longer SOAs, than those who have been less active. This association was significant even when controlling for a number of covariates including global cognitive function (MoCA), social connectedness score, and a series of sensory, health, and lifestyle factors. Thus, our data suggest that long-term physical activity behaviour is a noteworthy factor for multisensory function.

By demonstrating an association between multisensory perception and ongoing physical activity over a ten-year period, our findings extend previous cross-sectional studies that had indicated more precise multisensory processing [22] and greater benefit from multisensory stimulation [9] with higher physical activity. Our finding addresses the issue with cross-sectional findings that the association between multisensory processing and physical activity could be solely due to short-term factors, such as arousal, and supports the idea that either the association is due to the perceptual processes involved in being physically active, or, more generally, to better brain health in those who consistently exercise more. Note, however, that we controlled for performance on the MoCA. Given the positive association of higher physical activity on cognition [18, 23], these findings highlight the importance of maintaining a high level of physical activity for better brain health, including a specific effect on how the senses are integrated.

Interestingly, the result from the cross-sectional analysis (see Fig 4A) at wave 3 presented no significant association. This suggests that, in this cohort, only sustained or long-term physical activity is associated with the precision of temporal multisensory integration (unlike [9]). As such, IPAQ measured at a single time point may not be sensitive enough to determine a relationship between activity levels and multisensory perception concerning the temporal binding window. In turn, this may explain reported null relationships between IPAQ and SIFI in previous studies [22].

Another point refers to the overall difference in IPAQ between male and female participants, which mirrors recent findings on sex differences in SIFI susceptibility in the TILDA cohort [2]. Considering this fact (see S2 Fig), we conducted further analyses including the IPAQ Traj*SEX*SOA interaction with the model (S3 Fig and S5 Table). The results indicated that there was an interaction between the stable high activity trajectory class and SIFI at 230 ms SOA in female participants but not in male participants. In other words, a long-term level of high activity is associated with the precision of temporal multisensory integration mostly in females. This may indicate that differences between male and female participants in multisensory integration would emerge when the participants do have a low level of activity for a long time, or that less precise multisensory processing is a barrier to physical activity for females. However, as SIFI data prior to wave 3 are not available we cannot distinguish between these hypotheses. Hence, the association between the sex of the participant and multisensory integration is a matter of future investigation. Moreover, in this model, participants who are in the ‘Decreasing’ activity class showed higher SIFI susceptibility at 230 ms SOA than the reference class (i.e., ‘Stable [low]’ class). A decreasing physical activity trajectory may be likely to represent intrinsic, pathological issues such as emerging frailty and perturbed balance function, thus explaining its specific association with multisensory integration [16, 46].

The variance of our participants’ age should be noted in this study. Older adults (e.g., >75) might find it difficult to conduct moderate and vigorous exercise. While keeping active during all ages is essential, a number of studies support the idea that different amounts of physical activity are recommended for different age classes [47, 48], hence the active level might be a dynamic concept rather than static across the population. Moreover, although IPAQ was found to be a useful tool for assessing physical activity [36, 39, 49–51], a number of previous studies have noted problems with overestimation and underestimation of physical activity based on a subjective report in the IPAQ [52–54]. Accordingly, the use of more objective methods for measuring physical activity, such as wearable technology including an accelerometer, might be beneficial to investigate the association between multisensory perception and physical activity in more detail and across more targeted timescales.

### Limitation

Although this study helps elucidate and expand existing findings, in a large cohort, there are some limitations that must be considered. First, regardless of the large number of participants, the number of trials tested on the SIFI per participant had to be limited due to the time constraints of the TILDA protocol; there were two trials per six illusory conditions. This limits the range of analyses we can do, for example, learning is thought to be influential in how we perceive multisensory events [55], accordingly, we are unable to confirm whether the current effects would be anticipated if more trials or conditions were included. Also, the fact that our primary outcome measure is currently only available at wave 3 of the TILDA and that the physical activity trajectories were derived using five-time points is another limitation of our longitudinal analyses. As a result, it is challenging to determine the directionality or causality in the relationship between multisensory function and physical activity.

## Conclusion

In conclusion, we investigated the relationship between longitudinal trajectories of physical activity over ten years and multisensory integration (via the Sound-Induced Flash Illusion; SIFI) in 2,974 older adults. The precision by which auditory and visual events were integrated across time was influenced by the level of physical activity over a ten-year trajectory, such that those with stable high levels of activity showed more precise integration. These findings will help to elucidate the complex interplay between physical activity and multisensory integration in ageing and suggest that maintaining a high level of physical activity in older age can be beneficial for multisensory integration, which, in turn, is associated with cognition and mobility.

## Supporting information

S1 Fig Model comparisons. Lower Bayesian information criterion (BIC) indicates better model fitting.
(DOCX)

S2 Fig Distribution of participants with high activity level trajectory grouped by sex. (DOCX)

S3 Fig Longitudinal analysis of performance on the illusory 2B1F condition of the SIFI task with IPAQ trajectory with the sex interaction. (DOCX)

S1 Table Descriptive statistics for numerical (mean, SD) and categorical (count, %) variables per IPAQ trajectory class (“Increasing”, “Decreasing”, “Stable [low]”, and “Stable [high]”) at waves 1 and 3. Overall significant class effects are indicated by bold *p* values across variables.
(DOCX)

S2 Table Model comparisons. Lower BIC indicates better model fitting. For each model, the number of classes, log-likelihood, and percentage of participants in each of the classes have been shown.
(DOCX)

S3 Table Full results of the model predicting accuracy on 2B1F trials of the SIFI at the wave 3. (DOCX)

S4 Table Full results of the model predicting accuracy on 2B1F trials of the SIFI. (DOCX)

S5 Table Full results of the model predicting accuracy on 2B1F trials of the SIFI with the sex interaction. (DOCX)
