# Supplementary material for: Audio-visual integration is more precise in older adults with a high level of long-term physical activity
Source: PLoS One. 2023 Oct 4;18(10):e0292373. doi: 10.1371/journal.pone.0292373 (PMC10550131; doi:10.1371/journal.pone.0292373)
Supplement: S1 Fig — Lower Bayesian information criterion (BIC) indicates better model fitting. (DOCX) [file pone.0292373.s001.docx]

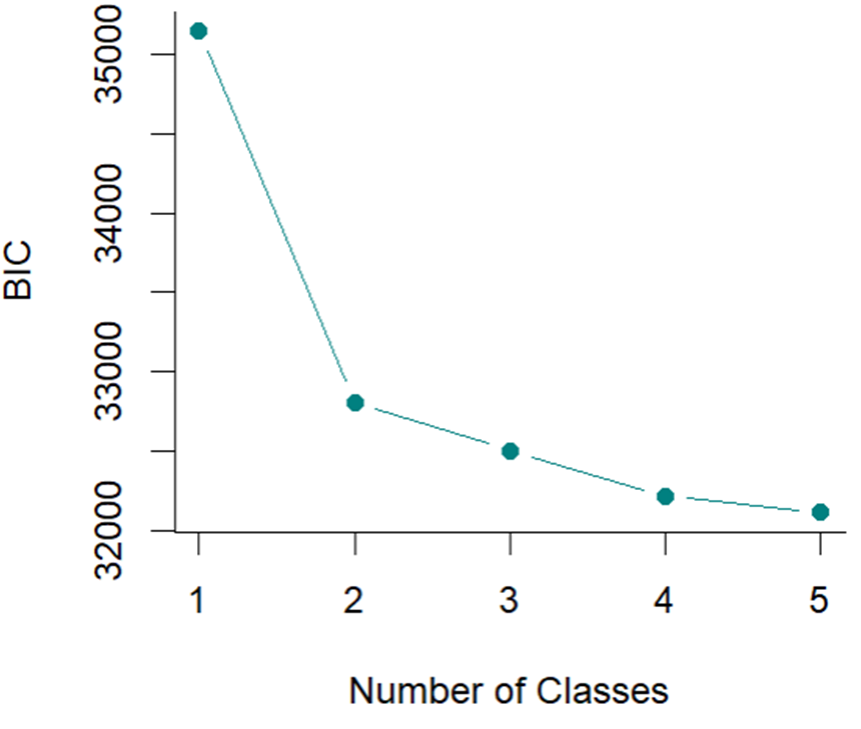


**S1 Fig.** **Model Comparisons.** Lower Bayesian information criterion (BIC) indicates better model fitting.
