# Supplementary figures and images for: Audio-visual integration is more precise in older adults with a high level of long-term physical activity
Source: PLoS One. 2023 Oct 4;18(10):e0292373. doi: 10.1371/journal.pone.0292373 (PMC10550131; doi:10.1371/journal.pone.0292373)

**
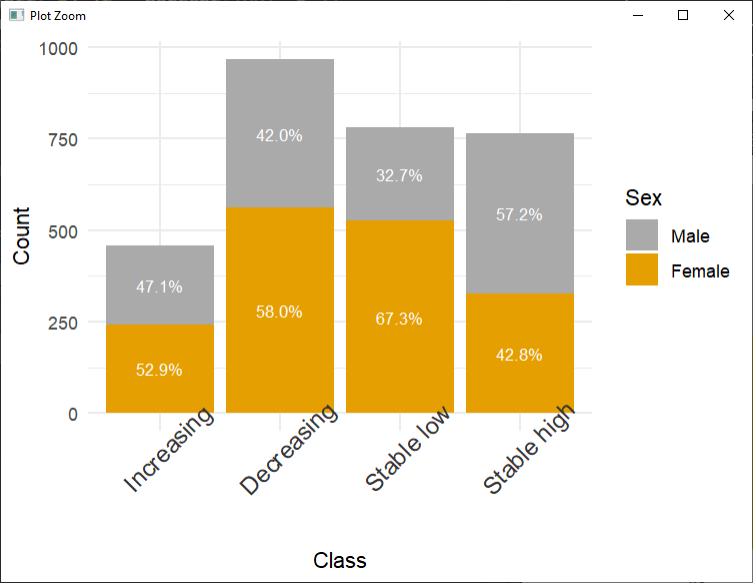
**

**S2 Fig.** **Distribution of participants with high activity level trajectory grouped by sex.**

Supplement: S2 Fig — (DOCX) [file pone.0292373.s002.docx]
