# Supplementary material for: Audio-visual integration is more precise in older adults with a high level of long-term physical activity
Source: PLoS One. 2023 Oct 4;18(10):e0292373. doi: 10.1371/journal.pone.0292373 (PMC10550131; doi:10.1371/journal.pone.0292373)
Supplement: S3 Fig — (DOCX) [file pone.0292373.s003.docx]

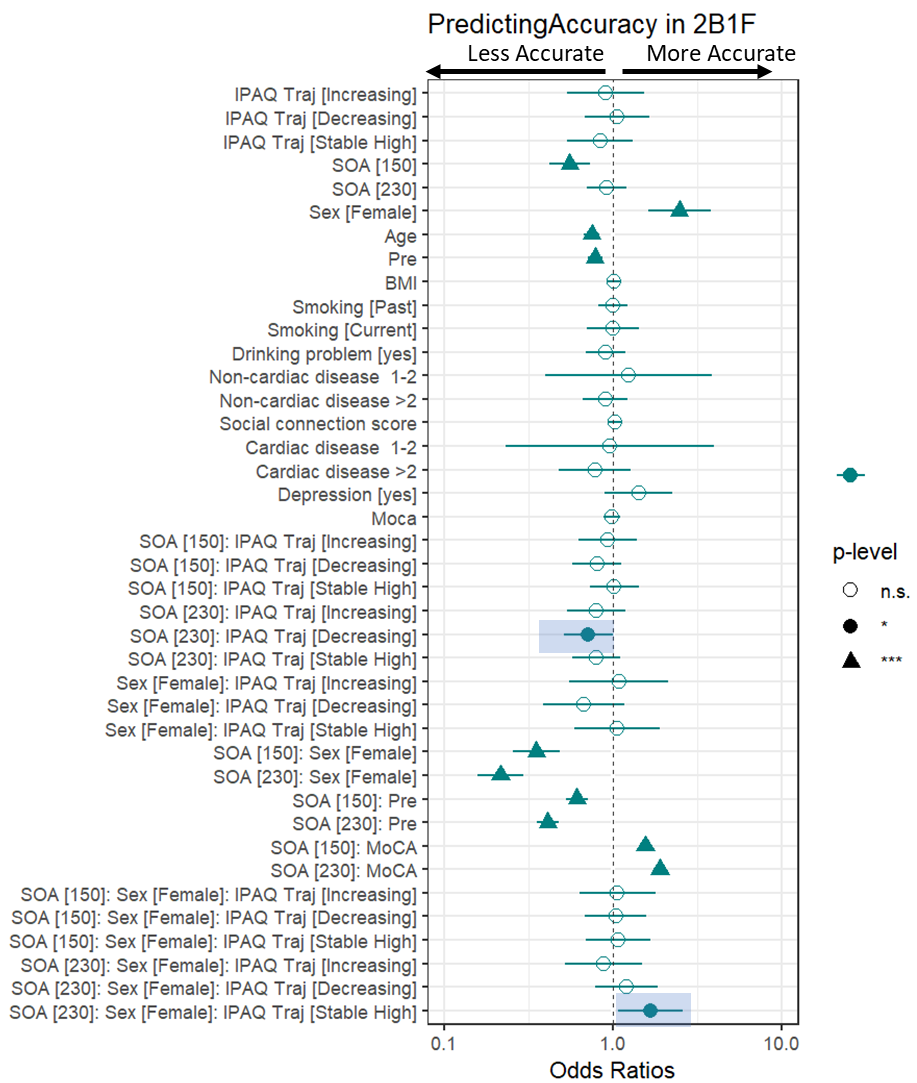


**S3 Fig.** Longitudinal analysis of performance on the illusory 2B1F condition of the SIFI task with IPAQ trajectory with the sex interaction.
