# Supplementary material for: Audio-visual integration is more precise in older adults with a high level of long-term physical activity
Source: PLoS One. 2023 Oct 4;18(10):e0292373. doi: 10.1371/journal.pone.0292373 (PMC10550131; doi:10.1371/journal.pone.0292373)
Supplement: S1 Table — Overall significant class effects are indicated by bold p values across variables. (DOCX) [file pone.0292373.s004.docx]

**S1 Table.** Descriptive statistics for numerical (mean, SD) and categorical (count, %) variables per IPAQ trajectory class (“Increasing”, “Decreasing”, “Stable [low]”, and “Stable [high]”) at waves 1 and 3. Overall significant class effects are indicated by bold *p* values across variables.

| IPAQ trajectory | | Increasing (n =496) | Decreasing (n =967) | Stable [low] (n =728) | Stable [high] (n =782) | p |
| --- | --- | --- | --- | --- | --- | --- |
| Age | Min | 50 | 50 | 50 | 50 | .001 |
|  | Max | 85 | 87 | 89 | 84 |  |
|  | Mean (sd) | 63.41 (7.03) | 64.98 (7.80) | 66.01 (8.29) | 63.02 (7.24) |  |
| BMI | Min | 17.70 | 17.04 | 16.83 | 17.93 | .61 |
|  | Max | 43.73 | 43.22 | 44.20 | 42.24 |  |
|  | Mean (sd) | 27.94 (4.41) | 28.20 (4.50) | 29.18 (5.02) | 27.71 (5.15) |  |
| Sex | Female (%) | 243 (53) | 561 (58) | 526 (67) | 328 (43) | <.001 |
|  | Male (%) | 216 (47) | 406 (42) | 256 (33) | 438 (57) |  |
| Education | Primary/none (%) | 64 (14) | 165 (17) | 133 (17) | 115 (15) | <.001 |
|  | Secondary (%) | 167 (36) | 369 (38) | 344 (44) | 302 (39) |  |
|  | Third/higher (%) | 228 (50) | 433 (45) | 305 (39) | 349 (46) |  |
| Smoking history | Never smoked (%) | 219 (48) | 457 (47) | 398 (51) | 367 (48) | <.001 |
|  | Past smoker (%) | 194 (42) | 418 (43) | 313 (40) | 333 (43) |  |
|  | Current smoker (%) | 46 (10) | 92 (10) | 71 (9) | 66 (9) |  |
| Heavy drinker | No (%) | 351 (71) | 745 (77) | 621 (85) | 586 (74) | <.001 |
|  | Yes (%) | 63 (13) | 126 (13) | 82 (11) | 102 (13) |  |
|  | No data (%) | 82 (16) | 96 (10) | 25 (3) | 70 (10) |  |
| MoCA score | Min | 16 | 9 | 11 | 15 | .79 |
|  | Max | 30 | 30 | 30 | 30 |  |
|  | Mean (sd) | 26.56 (2.70) | 26.06 (2.98) | 25.95 (3.09) | 26.49 (2.70) |  |
