# Supplementary material for: Audio-visual integration is more precise in older adults with a high level of long-term physical activity
Source: PLoS One. 2023 Oct 4;18(10):e0292373. doi: 10.1371/journal.pone.0292373 (PMC10550131; doi:10.1371/journal.pone.0292373)
Supplement: S2 Table — Lower BIC indicates better model fitting. For each model, the number of classes, log-likelihood, and percentage of participants in each of the classes have been shown. (DOCX) [file pone.0292373.s005.docx]

**S2 Table.** Model Comparisons. Lower BIC indicates better model fitting. For each model, the number of classes, log-likelihood, and percentage of participants in each of the classes have been shown.

| Model | Number of classes | loglik | BIC | %Class1 | %Class2 | %Class3 | %Class4 | %Class5 |
| --- | --- | --- | --- | --- | --- | --- | --- | --- |
| 1 | 1 | -17551.65 | 35151.29 | 100.00 |  |  |  |  |
| 2 | 2 | -16355.79 | 32807.56 | 47.41 | 52.59 |  |  |  |
| 3 | 3 | -16179.20 | 32502.36 | 30.23 | 34.40 | 35.38 |  |  |
| 4 | 4 | -16010.70 | 32213.35 | 15.43 | 32.51 | 26.29 | 25.76 |  |
| 5 | 5 | -15935.75 | 32111.43 | 20.32 | 25.49 | 4.85 | 24.88 | 24.48 |
