# Supplementary material for: Audio-visual integration is more precise in older adults with a high level of long-term physical activity
Source: PLoS One. 2023 Oct 4;18(10):e0292373. doi: 10.1371/journal.pone.0292373 (PMC10550131; doi:10.1371/journal.pone.0292373)
Supplement: S3 Table — (DOCX) [file pone.0292373.s006.docx]

**S3 Table.** Full results of the model predicting accuracy on 2B1F trials of the SIFI at the wave 3.

|  | Accuracy | | |
| --- | --- | --- | --- |
| Predictors | Odds Ratios | CI | p |
| (Intercept) | 1.43 | 0.76 – 2.70 | 0.266 |
| IPAQ level [Moderate] | 0.88 | 0.69 – 1.12 | 0.286 |
| IPAQ level [High] | 0.87 | 0.67 – 1.13 | 0.298 |
| SOA [150] | 0.28 | 0.25 – 0.33 | **<0.001** |
| SOA [230] | 0.35 | 0.31 – 0.41 | **<0.001** |
| Sex [Female] | 1.09 | 0.89 – 1.33 | 0.405 |
| Age | 0.75 | 0.68 – 0.83 | **<0.001** |
| PrePost [Pre] | 0.78 | 0.71 – 0.87 | **<0.001** |
| BMI | 1.01 | 0.92 – 1.11 | 0.891 |
| Smoker [Past] | 1.00 | 0.82 – 1.22 | 0.992 |
| Smoker [Current] | 0.98 | 0.69 – 1.40 | 0.917 |
| Alcohol consumption [Yes] | 0.92 | 0.70 – 1.20 | 0.530 |
| Education [Secondary] | 1.12 | 0.84 – 1.50 | 0.432 |
| Education [Tertiary] | 1.35 | 1.00 – 1.81 | **0.050** |
| Visual Acuity Score | 0.95 | 0.86 – 1.05 | 0.340 |
| Fair/poor hearing | 1.11 | 1.01 – 1.23 | **0.039** |
| Fair/poor vision | 0.96 | 0.87 – 1.06 | 0.475 |
| Chronic conditions [2+] | 1.16 | 0.38 – 3.59 | 0.794 |
| Chronic conditions [1] | 0.90 | 0.67 – 1.22 | 0.498 |
| Social connectedness score | 1.02 | 0.93 – 1.13 | 0.644 |
| Cardiovascular conditions [2+] | 0.94 | 0.23 – 3.89 | 0.933 |
| Cardiovascular conditions [1] | 0.78 | 0.48 – 1.27 | 0.312 |
| Depression [Yes] | 1.35 | 0.85 – 2.13 | 0.200 |
| 1B1F | 1.54 | 1.39 – 1.72 | **<0.001** |
| 2B0F | 0.91 | 0.83 – 1.01 | 0.068 |
| 0B2F | 0.70 | 0.52 – 0.99 | 0.125 |
| MoCA | 1.01 | 0.91 – 1.13 | 0.807 |
| IPAQ level [Moderate] * SOA [150] | 1.03 | 0.86 – 1.23 | 0.753 |
| IPAQ level [High] * SOA [150] | 1.00 | 0.82 – 1.22 | 0.985 |
| IPAQ level [Moderate] * SOA [230] | 0.93 | 0.77 – 1.11 | 0.409 |
| IPAQ level [High] * SOA [230] | 0.93 | 0.77 – 1.14 | 0.499 |
| SOA [150] * PrePost [Pre] | 0.61 | 0.52 – 0.71 | **<0.001** |
| SOA [230] * PrePost [Pre] | 0.41 | 0.36 – 0.48 | **<0.001** |
| SOA [150] * MoCA | 1.50 | 1.38 – 1.63 | **<0.001** |
| SOA [230] * MoCA | 1.81 | 1.66 – 1.97 | **<0.001** |
