# Supplementary material for: Audio-visual integration is more precise in older adults with a high level of long-term physical activity
Source: PLoS One. 2023 Oct 4;18(10):e0292373. doi: 10.1371/journal.pone.0292373 (PMC10550131; doi:10.1371/journal.pone.0292373)
Supplement: S4 Table — (DOCX) [file pone.0292373.s007.docx]

**S4** **Table.** Full results of the model predicting accuracy on 2B1F trials of the SIFI.

|  | Accuracy | | |
| --- | --- | --- | --- |
| Predictors | Odds Ratios | CI | p |
| (Intercept) | 1.59 | 0.84 – 3.03 | 0.156 |
| IPAQ trajectory class [Increasing] | 0.83 | 0.59 – 1.15 | 0.265 |
| IPAQ trajectory class [Decreasing] | 0.77 | 0.59 – 1.00 | 0.051 |
| IPAQ trajectory class [Stable high] | 0.69 | 0.52 – 0.92 | **0.013** |
| SOA [150] | 0.27 | 0.23 – 0.32 | **<0.001** |
| SOA [230] | 0.32 | 0.27 – 0.37 | **<0.001** |
| Sex [Female] | 1.08 | 0.89 – 1.32 | 0.429 |
| Age | 0.75 | 0.68 – 0.84 | **<0.001** |
| PrePost [Pre] | 0.78 | 0.71 – 0.87 | **<0.001** |
| BMI | 1.01 | 0.91 – 1.11 | 0.909 |
| Smoker [Past] | 1.01 | 0.82 – 1.23 | 0.959 |
| Smoker [Current] | 1.00 | 0.70 – 1.42 | 0.981 |
| Alcohol consumption [Yes] | 0.92 | 0.70 – 1.20 | 0.528 |
| Education [Secondary] | 1.12 | 0.84 – 1.50 | 0.445 |
| Education [Tertiary] | 1.36 | 1.01 – 1.83 | **0.044** |
| Visual Acuity Score | 0.95 | 0.86 – 1.05 | 0.320 |
| Fair/poor hearing | 1.11 | 1.00 – 1.23 | **0.041** |
| Fair/poor vision | 0.97 | 0.87 – 1.07 | 0.484 |
| Chronic conditions [2+] | 1.21 | 0.39 – 3.75 | 0.737 |
| Chronic conditions [1] | 0.90 | 0.66 – 1.22 | 0.495 |
| Social connectedness score | 1.02 | 0.93 – 1.13 | 0.640 |
| Cardiovascular conditions [2+] | 0.94 | 0.23 – 3.88 | 0.930 |
| Cardiovascular conditions [1] | 0.79 | 0.48 – 1.28 | 0.334 |
| Depression [Yes] | 1.39 | 0.88 – 2.19 | 0.163 |
| 1B1F | 1.54 | 1.38 – 1.72 | **<0.001** |
| 2B0F | 0.91 | 0.83 – 1.00 | 0.061 |
| 0B2F | 0.71 | 0.53 – 0.99 | 0.111 |
| MoCA | 1.02 | 0.91 – 1.14 | 0.770 |
| IPAQ trajectory class [Increasing] * SOA [150] | 1.11 | 0.87 – 1.43 | 0.402 |
| IPAQ trajectory class [Decreasing] * SOA [150] | 0.92 | 0.75 – 1.13 | 0.429 |
| IPAQ trajectory class [Stable high] * SOA [150] | 1.37 | 1.11 – 1.69 | **0.004** |
| IPAQ trajectory class [Increasing] * SOA [230] | 0.92 | 0.71 – 1.18 | 0.516 |
| IPAQ trajectory class [Decreasing] * SOA [230] | 0.94 | 0.77 – 1.15 | 0.534 |
| IPAQ trajectory class [Stable high] * SOA [230] | 1.46 | 1.18 – 1.80 | **<0.001** |
| SOA [150] * PrePost [Pre] | 0.61 | 0.52 – 0.71 | **<0.001** |
| SOA [230] * PrePost [Pre] | 0.41 | 0.35 – 0.48 | **<0.001** |
| SOA [150] * MoCA | 1.49 | 1.37 – 1.62 | **<0.001** |
| SOA [230] * MoCA | 1.80 | 1.65 – 1.96 | **<0.001** |
