# Supplementary material for: Audio-visual integration is more precise in older adults with a high level of long-term physical activity
Source: PLoS One. 2023 Oct 4;18(10):e0292373. doi: 10.1371/journal.pone.0292373 (PMC10550131; doi:10.1371/journal.pone.0292373)
Supplement: S5 Table — (DOCX) [file pone.0292373.s008.docx]

**S5 Table.** Full results of the model predicting accuracy on 2B1F trials of the SIFI with the sex interaction.

|  | Accuracy | | |
| --- | --- | --- | --- |
| Predictors | Odds Ratios | CI | p |
| (Intercept) | 0.92 | 0.46 – 1.86 | 0.826 |
| IPAQ trajectory class [Increasing] | 0.90 | 0.53 – 1.52 | 0.688 |
| IPAQ trajectory class [Decreasing] | 1.05 | 0.67 – 1.64 | 0.822 |
| IPAQ trajectory class [Stable high] | 0.84 | 0.54 – 1.30 | 0.431 |
| SOA [150] | 0.55 | 0.42 – 0.72 | **<0.001** |
| SOA [230] | 0.91 | 0.70 – 1.19 | 0.506 |
| Sex [Female] | 2.47 | 1.61 – 3.79 | **<0.001** |
| Age | 0.75 | 0.67 – 0.83 | **<0.001** |
| PrePost [Pre] | 0.78 | 0.70 – 0.87 | **<0.001** |
| BMI | 1.01 | 0.91 – 1.11 | 0.898 |
| Smoker [Past] | 1.00 | 0.82 – 1.22 | 0.987 |
| Smoker [Current] | 1.00 | 0.70 – 1.43 | 0.991 |
| Alcohol consumption [Yes] | 0.90 | 0.69 – 1.19 | 0.472 |
| Education [Secondary] | 1.11 | 0.83 – 1.49 | 0.480 |
| Education [Tertiary] | 1.34 | 0.99 – 1.80 | 0.059 |
| Visual Acuity Score | 0.95 | 0.86 – 1.05 | 0.277 |
| Poor hearing | 1.12 | 1.01 – 1.23 | **0.032** |
| Fair/poor vision | 0.96 | 0.87 – 1.07 | 0.473 |
| Chronic conditions [2+] | 1.24 | 0.40 – 3.85 | 0.713 |
| Chronic conditions [1] | 0.90 | 0.66 – 1.22 | 0.479 |
| Social connectedness score | 1.02 | 0.93 – 1.13 | 0.656 |
| Cardiovascular conditions [2+] | 0.96 | 0.23 – 3.99 | 0.950 |
| Cardiovascular conditions [1] | 0.78 | 0.48 – 1.28 | 0.325 |
| Depression [Yes] | 1.42 | 0.89 – 2.25 | 0.139 |
| 1B1F | 1.54 | 1.39 – 1.72 | **<0.001** |
| 2B0F | 0.91 | 0.83 – 1.00 | 0.052 |
| 0B2F | 0.72 | 0.53 – 1.00 | 0.075 |
| MoCA | 0.99 | 0.88 – 1.10 | 0.821 |
| IPAQ trajectory class [Increasing] * SOA [150] | 0.93 | 0.62 – 1.38 | 0.703 |
| IPAQ trajectory class [Decreasing] * SOA [150] | 0.80 | 0.58 – 1.12 | 0.200 |
| IPAQ trajectory class [Stable high] * SOA [150] | 1.02 | 0.73 – 1.41 | 0.922 |
| IPAQ trajectory class [Increasing] * SOA [230] | 0.80 | 0.53 – 1.18 | 0.260 |
| IPAQ trajectory class [Decreasing] * SOA [230] | 0.71 | 0.51 – 0.99 | **0.043** |
| IPAQ trajectory class [Stable high] * SOA [230] | 0.80 | 0.57 – 1.11 | 0.175 |
| IPAQ trajectory class [Increasing] * Sex [Female] | 1.08 | 0.55 – 2.12 | 0.828 |
| IPAQ trajectory class [Decreasing] * Sex [Female] | 0.67 | 0.39 – 1.17 | 0.162 |
| IPAQ trajectory class [Stable high] * Sex [Female] | 1.06 | 0.59 – 1.90 | 0.858 |
| SOA [150] * Sex [Female] | 0.35 | 0.25 – 0.48 | **<0.001** |
| SOA [230] * Sex [Female] | 0.21 | 0.16 – 0.29 | **<0.001** |
| SOA [150] * PrePost [Pre] | 0.61 | 0.52 – 0.71 | **<0.001** |
| SOA [230] * PrePost [Pre] | 0.41 | 0.35 – 0.48 | **<0.001** |
| SOA [150] * MoCA | 1.55 | 1.42 – 1.68 | **<0.001** |
| SOA [230] * MoCA | 1.89 | 1.74 – 2.06 | **<0.001** |
| (IPAQ trajectory class [Increasing] * SOA [150]) * Sex [Female] | 1.06 | 0.63 – 1.78 | 0.822 |
| (IPAQ trajectory class [Decreasing] * SOA [150]) * Sex [Female] | 1.03 | 0.68 – 1.58 | 0.879 |
| (IPAQ trajectory class [Stable high] * SOA [150]) * Sex [Female] | 1.07 | 0.69 – 1.67 | 0.752 |
| (IPAQ trajectory class [Increasing] * SOA [230]) * Sex [Female] | 0.88 | 0.52 – 1.49 | 0.633 |
| (IPAQ trajectory class [Decreasing] * SOA [230]) * Sex [Female] | 1.20 | 0.78 – 1.83 | 0.406 |
| (IPAQ trajectory class [Stable high] * SOA [230]) * Sex [Female] | 1.65 | 1.06 – 2.57 | **0.015** |
